# Functional instant noodle formulation for emergency conditions: Sensory and stability characteristics

**DOI:** 10.1002/fsn3.4062

**Published:** 2024-03-05

**Authors:** Farboud Shahabinejad, Maryam Ghorbani, Sepideh Abbaszadeh, Mohammad Nejatian, Maryam Taghdir

**Affiliations:** ^1^ Student Research Committee Baqiyatallah University of Medical Sciences Tehran Iran; ^2^ Department of Pharmacology and Toxicology, Faculty of Pharmacy Baqiyatallah University of Medical Sciences Tehran Iran; ^3^ Health Research Center, Life Style Institute Baqiyatallah University of Medical Sciences Tehran Iran; ^4^ Department of Nutrition and Food Hygiene, Faculty of Health Baqiyatallah University of Medical Sciences Tehran Iran

**Keywords:** emergency, noodle, shelf‐life, tactical ration

## Abstract

The aim of this study was to evaluate the shelf‐life and sensory characteristics of a functional instant noodle preparation designed to be used in emergencies as a tactical ration. Instant noodles were selected for their global acceptability and ease of preparation. In this study, semolina flour was used as the main ingredient, and soy protein isolate was added to increase the protein content. Additionally, green tea and beef tallow were incorporated to decrease the likelihood of oxidation. Carboxymethyl cellulose was added to increase the porosity and water absorption of the dry noodles. Spirulina powder was used as a dressing for the final product before serving to increase the nutritional value and provide the consumer with the required vitamins and minerals of the day. Physical, chemical, and organoleptic properties were assessed at multiple timepoints in a 120‐day period to perform an accelerated shelf‐life test by determining their critical moisture content and moisture sorption isotherm curves at 30, 45, and 55°C. The shelf‐life of the product was evaluated to be 1197.28 days at 30°C and 75% relative humidity in aluminum pouches. In conclusion, the product is shelf‐stable at room temperature and is recommended to be stored and used in disaster conditions such as earthquakes, floods, and wars.

## Graphical abstract

A mixture of semolina flour, soy protein isolate, carboxymethyl cellulose, baking powder, and green tea can be used to form a noodle dough to be fried in tallow, which can then be used as a tactical ration for disasters. The tested product has a shelf‐life of more than 3 years in accelerated shelf‐life calculations based on water activity and microbial growth.

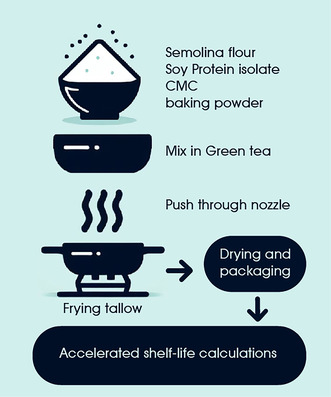

## 1 INTRODUCTION

Some food products are precisely designed for delicate conditions such as wars or disasters (Vijaya Rao, 2012). They're not designed to taste better or to persuade the customer to keep purchasing the product, but to make them survive that particular condition. This category of product, tactical rations, is one of the most strategically needed items in those conditions (Revista Panamericana de Salud Pública, 2000; World Health Organization, 2004). Usually, these products are stockpiled by governments, armies, or helping organizations to be used in conditions where food cannot be served or cooked (World Health Organization, 2004). Armies use these rations to increase the maneuverability of their soldiers when they need to move fast or have to survive an attack on food sources. Striking forces such as commandos benefit from the rations where there is no time to cook, and they have to carry as many calories as possible to endure a 3–5 day operation by themselves (Booth, 2003; Forbes‐Ewan et al., 2016). Such requirements lead to the development of various rations designed for specific conditions and locations, from floods to wars, and mountains to the seas (Hamilton, 2003). During the process of respiration, the body loses some of its electrolytes, such as sodium, potassium, calcium, and magnesium (Feagans et al., 2010). Normally, a normal diet consisting of ordinary foods with added vegetables contains enough vitamins and minerals for a normal routine of life. On the other hand, amid a disaster, battle, or even a hike, there is not enough time and maneuverability to add fruits and vegetables to the dish (Seal & Thurstans, 2013). In those times, the packed food must cover the necessary vitamins and minerals singlehandedly, as if no other food items were available to the consumer [Hu & Jacobsen, 2016; Institute of Medicine; Food and Nutrition Board; Committee on Optimization of Nutrient Composition of Military Rations for Short‐Term, High‐Stress Situations; Committee on Military Nutrition Research, 2005Institute of Medicine (US) Subcommittee on Technical Specifications for a High‐Energy Emergency Relief Ration, 2002; Revista Panamericana de Salud Pública, 2000]. We formulated a functional product for the same purpose. This product is designed to maintain an adult survivor of a disaster or a soldier for the majority of the day. One of the problems in the formulation of tactical rations is the different palates of different nations (Research and Marriott, 1995). A tasteful dish in Asia may look impossible to eat for an American, and vice versa (Feeney et al., 2009). To develop a globally well‐liked formulation, instant noodles were selected. Instant noodles are popular around the world, and the palates of many people have already become familiar with them. Another benefit is the low price and the simplicity of production. Instant noodles can be easily flavored, and the preparation is quite simple.

As will be discussed later, competing products are either more expensive to make, less palatable, or lack important nutrients. The incorporated ingredients are well balanced to not only maintain blood glucose over a long period but also to help consumers stay sharp and in a calm state.

In this study, an instant noodle formulation is discussed, with composition alterations to extend its shelf‐life and adjust its nutritional value to be used in disaster conditions.

## 2 MATERIALS AND METHODS

### 2.1 Raw materials

All the required ingredients, such as semolina flour (Khousheh Fars Co., Iran, 16% moisture content 0.5% ash), baking powder (Golestan Co., Iran), beef tallow (Salemin Co., Iran, 49.8% saturated fat, 41.8% monounsaturated fat, 4% polyunsaturated fat), green tea (Golestan Co., Iran, dry leaves, 11% moisture content), carboxymethyl cellulose (CMC) (BalmiLife Co., Iran, 5.7% moisture content, 22.1% ash), and soy protein isolate (BehTaam powder Co., Iran, Alborz, 91% protein, 6.2% moisture content) were procured from the local market. Spirulina powder (OrganicAlgae Co., Shiraz, Iran, 58% protein, 6.5% fat, 5.5% moisture content, 7.1% ash) was also purchased to be used as a dressing for the final product. The required salts for water activity determination and laboratory materials were purchased from Sigma Aldrich, USA. The control sample was a commercial instant noodle preparation (Noodelite, meat flavor, Iran).

### 2.2 Sample preparation

**FIGURE 1:**
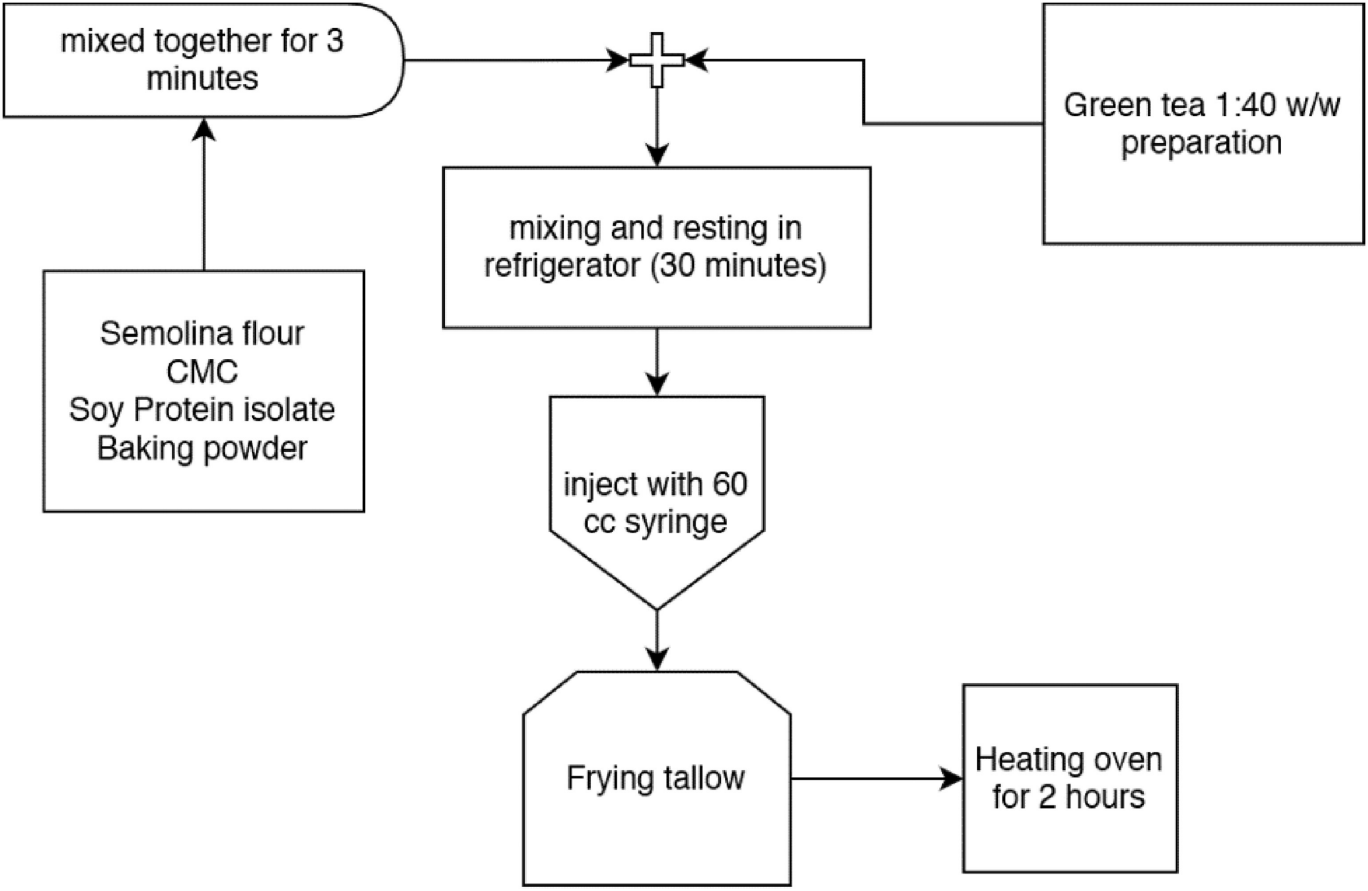
Sample preparation flowchart.

To produce the noodle, 500 g of semolina flour, 25 g of baking powder, 100 g of CMC, and 50 g of soy protein isolate were mixed using a mixer (ParsKhazar, OmegaPlus, Iran) for 3 min at a speed of 90 rpm. Green tea extract was prepared from dried tea leaves. For this purpose, the ground tea powder and distilled water (1:40 w/w) were stirred in an Erlenmeyer flask in a shaking water bath (Memmert, Germany) at 100°C for 15 min. Green tea extract was then added to the prepared mixture of noodles and mixed manually for 3 min. After resting for 10 min, the dough was mixed in the mixer at a speed of 180 rpm for 15 min to obtain a homogeneous dough. Meanwhile, the beef tallow was put in the frying container on the stove for 5 min to be melted first and to reach the boiling point. The dough was kept in the refrigerator for 30 min before being injected from 60 cc syringes into the frying tallow to be fried for 4 min until the color changed to brown. The fried noodles were then transferred to a preheated oven (80°C) for 2 h to dry. Each sample was then packed in aluminum pouches that were later sealed by indirect heat (iron) and marked for later tests. The pouches were put in boiling water for 30 min to get pasteurized. The process is depicted in Figure 1. For serving, the dry noodles were put in boiling water for 5 min (Hou, 2010), and then Spirulina powder was added to the cooked noodles.

### 2.3 Determination of chemical composition

Samples were stored at different temperatures (30, 45, and 55°C) to establish the stability curve. The total microbial count was calculated based on the Prescott et al. method (Prescott et al., 2001). Ash content was determined by the method described by Mortensen et al. (1989). Protein content was measured by the Kjeldahl method (Jiang et al., 2014). Total carbohydrate was analyzed by the phenol–sulfuric acid method (Nielsen, 2010). Using concentrated sulfuric acid, all classes of carbohydrates were converted into monosaccharides, which ultimately form yellowish compounds in the presence of 80% (wt/wt) phenol, which were assessed by spectrophotometery. The total fat content was evaluated by the Soxhlet method (Manirakiza et al., 2001). The pH measurements of the cooked noodles were performed by a calibrated pH meter (Clean, pH 500, Taiwan).

### 2.4 Determining shelf stability using organoleptic properties

To evaluate the stability of the noodle formulation, samples were stored at different temperatures (30, 45, and 55°C) for up to 120 days. The samples were evaluated at different time intervals (0, 30, 60, 90, and 120 days) for acceptability of physical and organoleptic properties by a panel of 15 trained judges (Fuad & Prabhasankar, 2012), comprising healthcare staff and regular civilians. The judges were trained to use the 5‐point scale for taste, odor, color, and texture of each sample according to ISO 6658:2017. The prepared noodles were served on tiny plates with Spirulina powder dressing. The judges were asked to evaluate the noodles on the basis of their acceptance of organoleptic properties on a 5‐point hedonic scale, which ranged from 1 (disliked very much) to 5 (liked very much) for each of the properties.

Each sample was put in boiling water for 5 min, and Spirulina powder was added before serving as a dressing. Organoleptic testing and water content analysis were conducted every week for a total of 12 weeks (Sarofa & Anggreini, 2019).

The evaluated physical and organoleptic properties included color, texture, taste, and odor. The critical moisture content of dry noodles was determined by storing the product without packaging at room temperature.

### 2.5 Determining shelf stability using relative humidity

The shelf stability of the noodles was evaluated by determining their equilibrium moisture content (*M*_e_) at different relative humidity (RH) levels based on the method of Sefrienda et al. (2022). This is because the moisture content of a food product can affect its shelf life, as it can lead to microbial growth, chemical reactions, and physical changes that can deteriorate the quality of the product over time.

### 2.6 Determination of equilibrium moisture

To determine the *M*_e_ at different RH levels, a sample of the food product was placed in a porcelain dish and stored in a jar filled with a saturated salt solution. Saturated solutions were LiCl, MgCl_2_, K_2_CO_3_, Mg(NO_3_)_2_, NaNO_2_, NaCl, KCl, and K_2_SO_4_ to maintain a constant RH level inside the jar, which allows for accurate measurement of the *M*_e_ of the food product. The dish was weighed periodically until a constant weight was achieved, indicating that the sample had reached equilibrium with the RH level inside the jar. The RH values of various salt solutions are mentioned in Table 1.

**TABLE 1:** Saturated salt solutions and their relative humidity percentages.

Saturated salt solution	Aw	RH (%) at 30°C	Moisture content (%)
LiCl	0.11	11.3	8.2
MgCl_2_	0.32	32.44	12.3
K_2_CO_3_	0.43	43	13.1
Mg(NO_3_)_2_	0.51	51.4	12.8
NaNO_2_	0.73	73.14	20.3
NaCl	0.75	75	25.1
KCl	0.84	83.62	37.3
K_2_SO_4_	0.92	92.31	52.5

The resulting data on *M*_e_ and RH were then used to create a smooth MSI (moisture sorption isotherm) curve using the GAB (Guggenheim–Anderson–de Boer) isotherm model. This model is commonly used to represent the sorption behavior of food products and can provide valuable information on the water activity (*a*_w_) of the food product, which is another important factor in determining its shelf stability (Putri et al., 2021; Sirpatrawan, 2009).

GAB equations:

$$\ln\left[\frac{M_{e}-M_{i}}{M_{e}-M_{c}}\right]=\left(\frac{k}{x}\right)\left(\frac{A}{W_{s}}\right)\left(\frac{P_{0}}{b}\right)\theta ,\text{and}\;\frac{M}{M_{0}}=\frac{C∙K∙a_{w}}{\left(1-K∙a_{w}\right)\left(1-K∙a_{w}+C∙K∙a_{w}\right)}$$

*b* = slope of the MSI curve.

*θ* = shelf‐life (days).

*P*_0_ = vapor pressure of pure water (mmHg).

*M*_0_ = monolayer value.

*C* and *K* are calculated constants.

### 2.7 MSI curve

By analyzing the MSI curve, the critical *a*_w_ levels, at which microbial growth and other deteriorative processes begin to occur, can be determined to establish appropriate storage conditions and shelf life for the food product. Overall, the use of relative humidity and saturated salt solutions in evaluating the shelf stability of food items such as noodles provides valuable insights into their moisture content and *a*_w_ levels, which are critical factors in ensuring their quality and safety over time.

### 2.8 Statistical analysis

All measurements were repeated three times to ensure reproducibility. Data obtained from the stability and shelf life studies were analyzed using statistical software (Microsoft Excel 2019 and Graphpad Prism 10).

## 3 RESULTS AND DISCUSSION

Tactical rations are valuable products that are used in times of disaster or war (Forbes‐Ewan et al., 2016; Montain & Young, 2003). To prepare a palatable dish for different cultures that also contains the essential micro‐ and macronutrients for disaster conditions, an instant noodle was formulated with multiple alterations, such as replacing traditional noodle ingredients with semolina flour, green tea, and beef tallow, and adding Spirulina powder as a dressing to the dish. The purpose of this study was to evaluate for how long the product is shelf‐stable so that policymakers can act accordingly.

### 3.1 Chemical components

The nutritional components of the formulation after preparation were analyzed. Each 100 g of the sample contained 443 KCalories, which consisted of 52.5% carbohydrates, 17.3% protein, 18.2% fat, and 1.1% ash. The pH of the final product at day 0 was 6.7.

In the process of formulation, wheat flour was replaced with semolina flour to increase the protein content of the final product, similar to the work of Thuy et al. (2020). The high amount of fiber and protein in the semolina flour theoretically would result in a low glycemic index, which can be extremely crucial in environments where food accessibility is limited (Hu & Jacobsen, 2016; Institute of Medicine; Food and Nutrition Board; Committee on Optimization of Nutrient Composition of Military Rations for Short‐Term, High‐Stress Situations; Committee on Military Nutrition Research, 2005). The downside is the brownish color of the final product.

### 3.2 Shelf‐life measures

A disaster condition requires the stockpiled rations to be shelf‐stable for an extended period compared to routine food products. Several studies have been dedicated to evaluating the best means of accelerated shelf‐life stability calculation (Bharath Kumar & Prabhasankar, 2018; Byun et al., 2010; Darsch & Moody, 2009; Ekafitri et al., 2021; González et al., 2017; Khan et al., 2014; Mitrevski et al., 2016; Putri et al., 2021; Sefrienda et al., 2022; Sirpatrawan, 2009; Tuersuntuoheti et al., 2019; Yang et al., 1997; Zhang et al., 2016). Researches have shown that water activity has been among the most preferred methods of shelf‐life estimation for noodles and similar products (Li et al., 2011; Mitrevski et al., 2016; Putri et al., 2021; Sefrienda et al., 2022; Sirpatrawan, 2009). Similar to the method used by Sefrienda et al. (2022), organoleptic tests were also used in the shelf‐life determination of the noodles, as well as the water activity‐based method.

### 3.3 Shelf‐life estimation based on organoleptic properties

**FIGURE 2:**
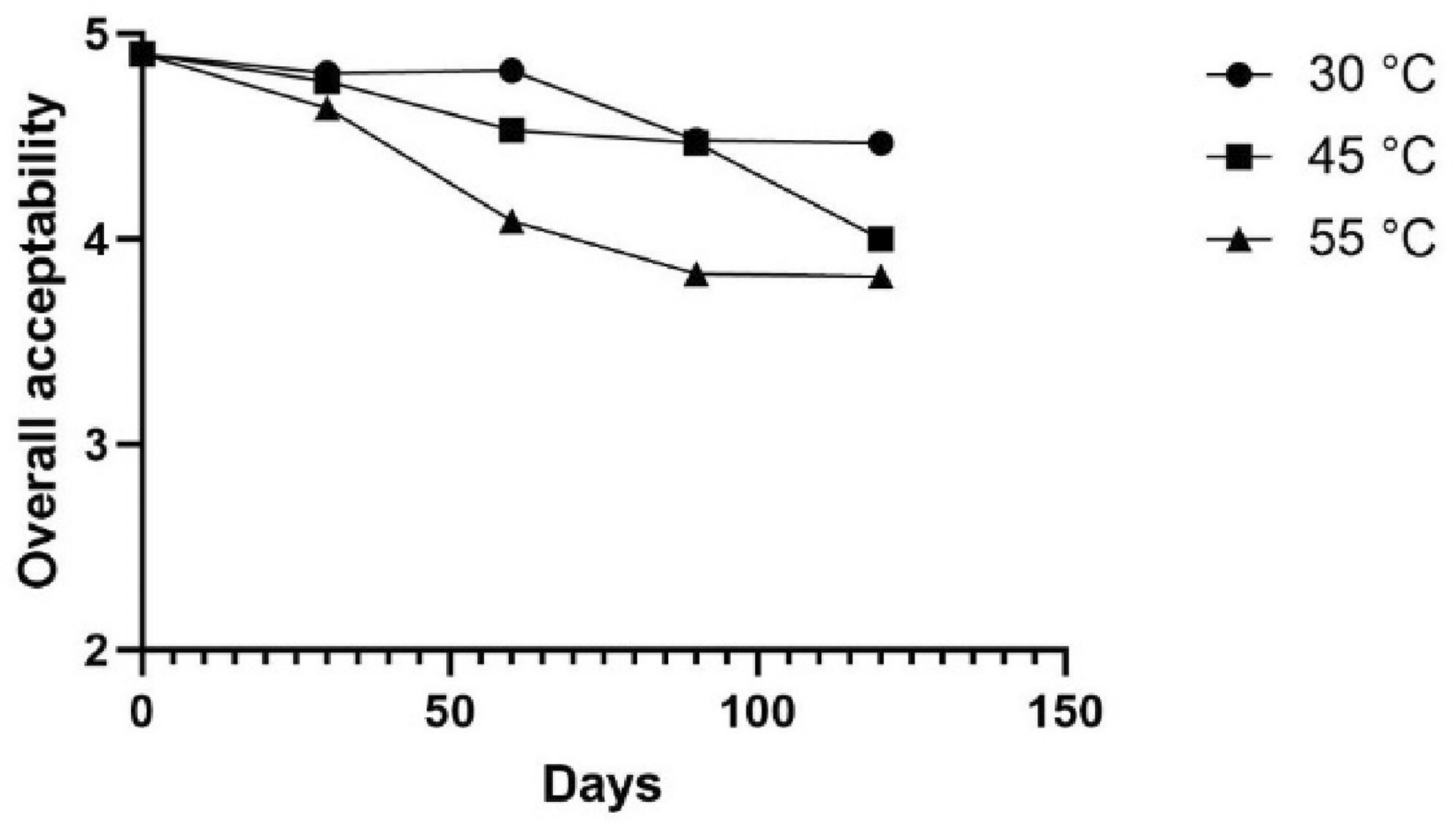
Hedonic scores of the samples.

**FIGURE 3:**
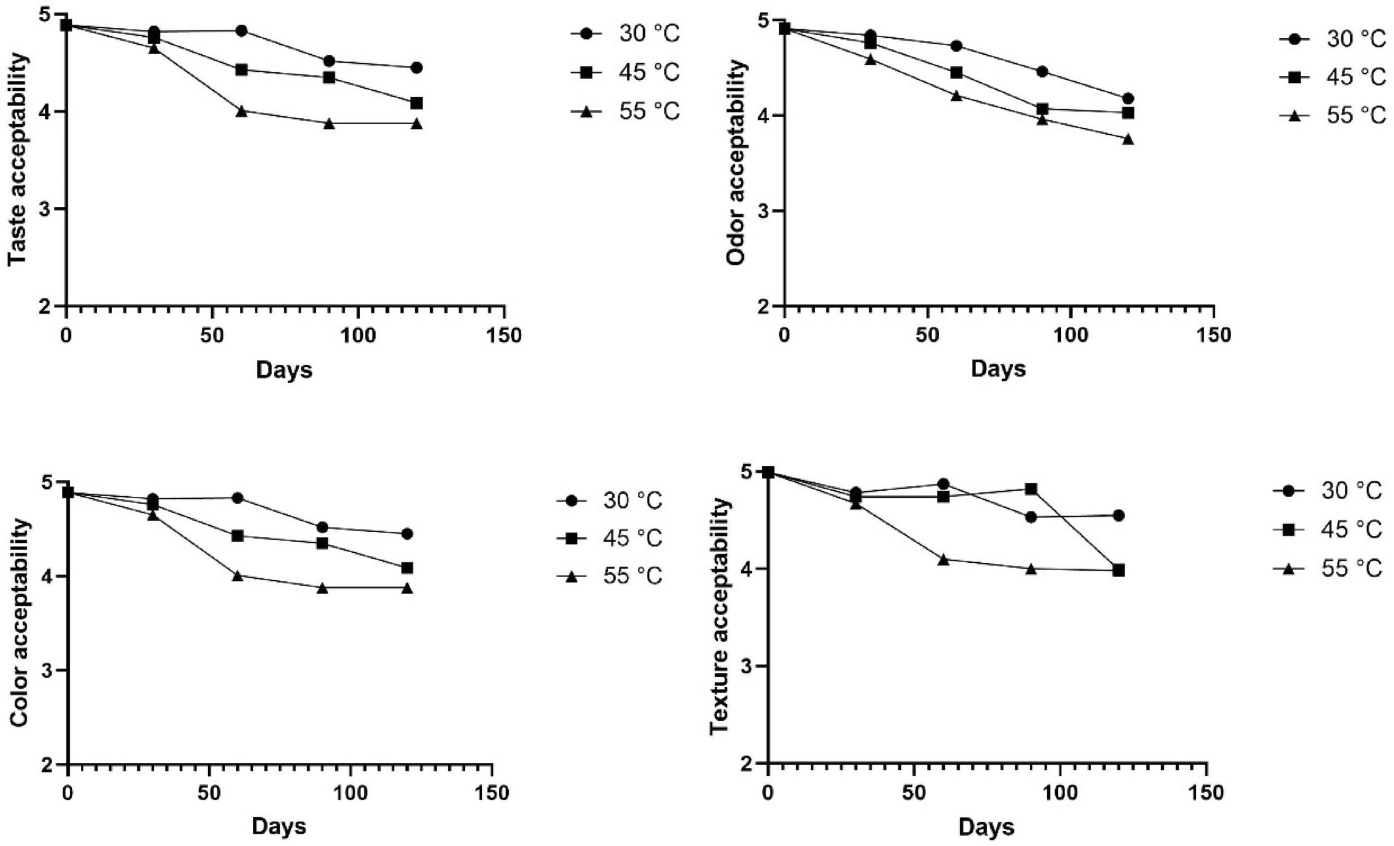
Organoleptic test results of dry noodles.

Organoleptic tests were not only used as a measure of shelf‐life stability, but also to test the palatability of the product. The mean of each criteria score was calculated to obtain the overall organoleptic score of the product on each day of storage at 30, 45, or 55°C (Figures 2 and 3). The means of scores were plotted in a curve to obtain each temperature's exponential equation for the shelf‐stability of the product.

The critical score of organoleptic unacceptability was set to 2 (disliked moderately). According to the obtained equations, the estimated shelf‐life of the product stored at 30, 45, and 55°C was calculated to be 1389, 648, and 372 days, respectively.

The organoleptic stability results showed that the noodle formulation was stable at all tested temperatures for up to 120 days. The taste, color, texture, and odor were deemed acceptable by the judges during the storage period. The increase in the storage temperature correlated with the acceptance scores, as anticipated. On the other hand, the overall acceptance of the product would decrease with an increase in its water content (Sarofa & Anggreini, 2019; Sefrienda et al., 2022).

### 3.4 Shelf‐life estimation based on water activity

Within 24 h after production, the initial moisture content of the product (*M*_i_) was evaluated according to AOAC to be 12.6%. The solid weight (*W*_s_) of a single serving of the product was 215 g. The packaging permeability $\left(\frac{k}{x}\right)$ of the aluminum packaging was estimated to be 0.00386 g H_2_O/m^2^ hari mmHg, the vapor pressure of pure water at 30°C was 31.80 mmHg, and the surface area (*A*) of the packaging was estimated to be 0.09 m^2^ (Ekafitri et al., 2021).

The crunchiness of dry noodle products was an important feature. It was supposed that the decrease in crunchiness leads to product damage and a decline in consumer satisfaction (Sarofa & Anggreini, 2019; Sefrienda et al., 2022). The crunchiness of dry noodles was determined by how easily the product could be broken. A score of 2 on a scale of 1–5 was considered critical in terms of water content, as it indicated that the product became slightly harder to break.

**FIGURE 4:**
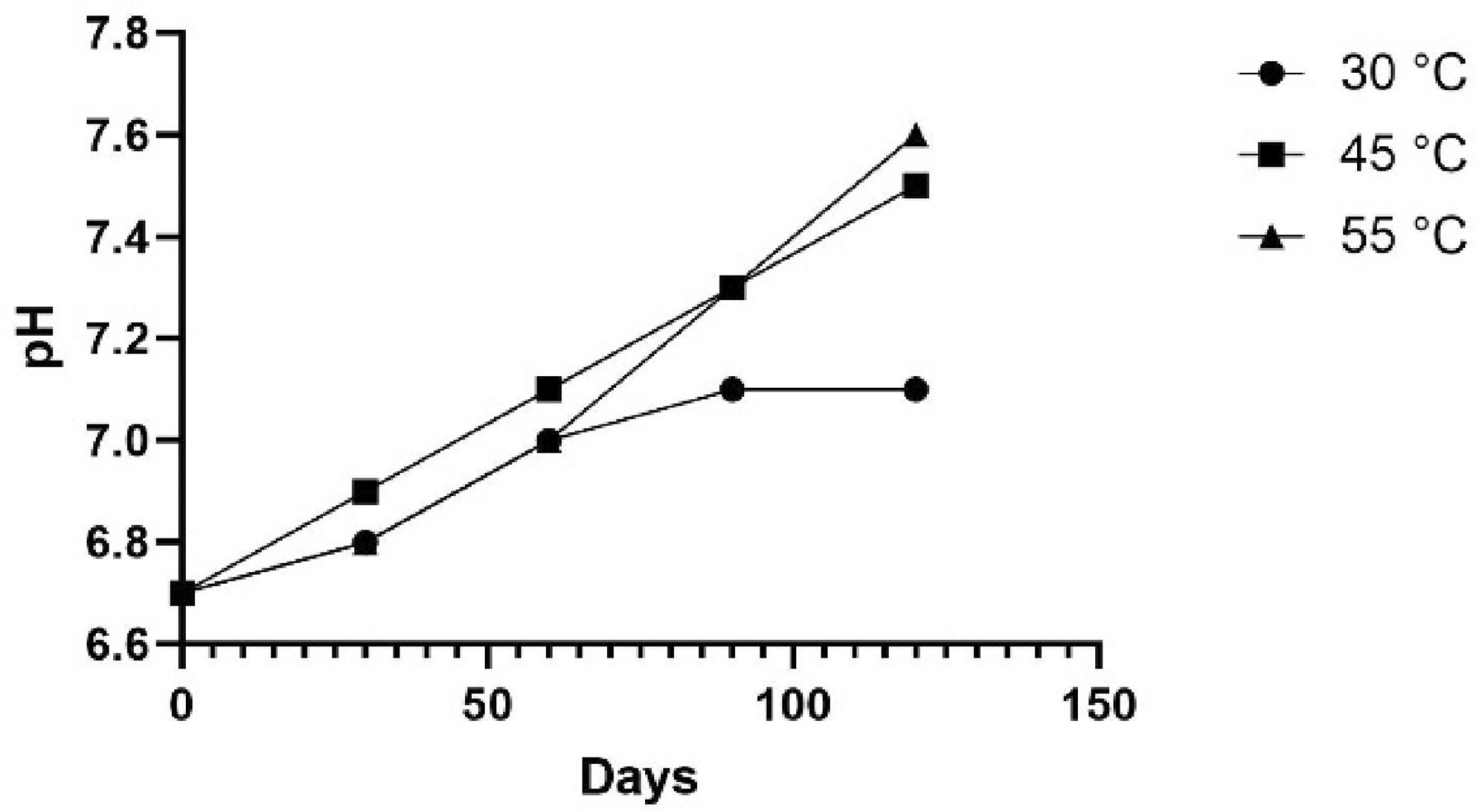
Changes in pH measurements of the final product.

The data on water content and panelists' preference scores for texture parameters were used to create a linear regression equation. By plotting the rejection score of 2 into the equation, the critical water content of the product was determined to be 13.7%. The changes in pH measurements of the final product are also depicted in Figure 4.

**FIGURE 5:**
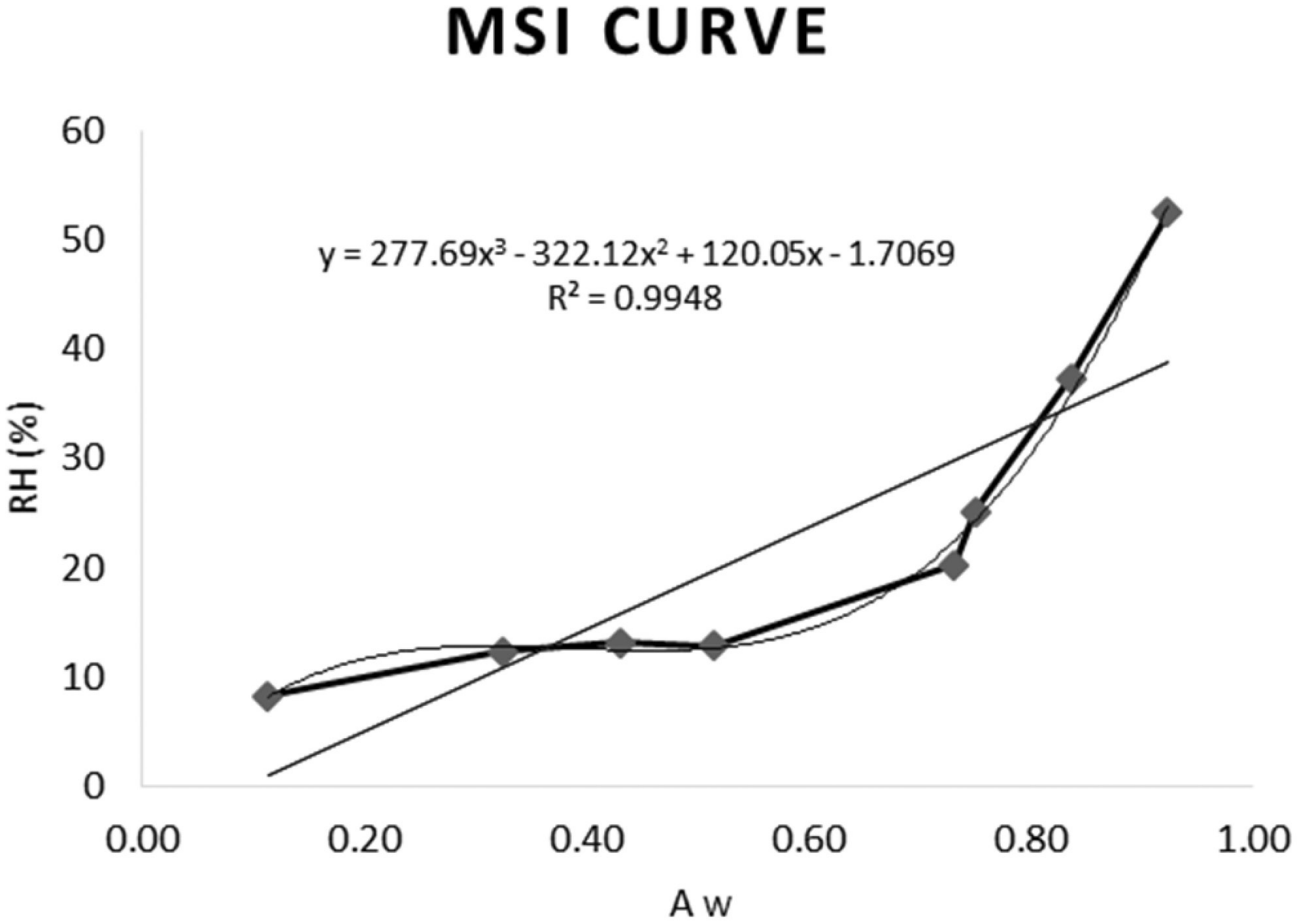
Moisture sorption isotherm (MSI) curve of dry noodles.

The equilibrium moisture content (*M*_e_) was calculated by putting the dry noodles in a porcelain dish placed in a jar filled with several saturated salt solutions, resulting in different relative humidity (RH) percentages correlated with higher water activities (*a*_w_) (Figure 5).

**FIGURE 6:**
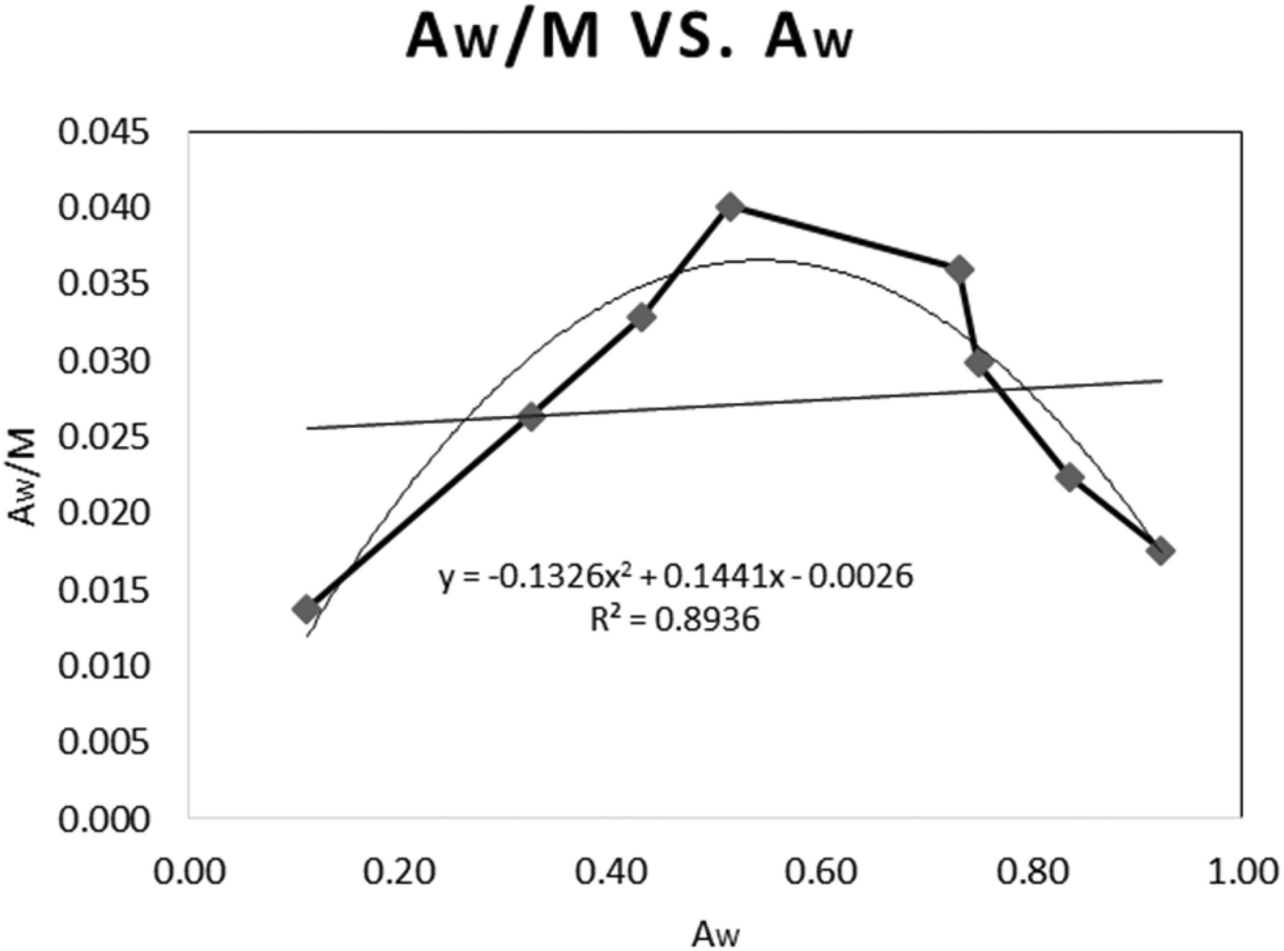
Equilibrium moisture content plotted against water activity.

To be used in the GAB equation, the moisture sorption isotherm (MSI) curve of the product was plotted using the calculated moisture contents of the product within each relative humidity. Furthermore, the critical moisture content and the initial moisture contents were put in the equation to calculate their correlation *a*_w_. According to the plot's equation (Figure 6), *K* was calculated to be 0.9054, *C* was 63.2137, and *M*_0_ was 6.72%.

**FIGURE 7:**
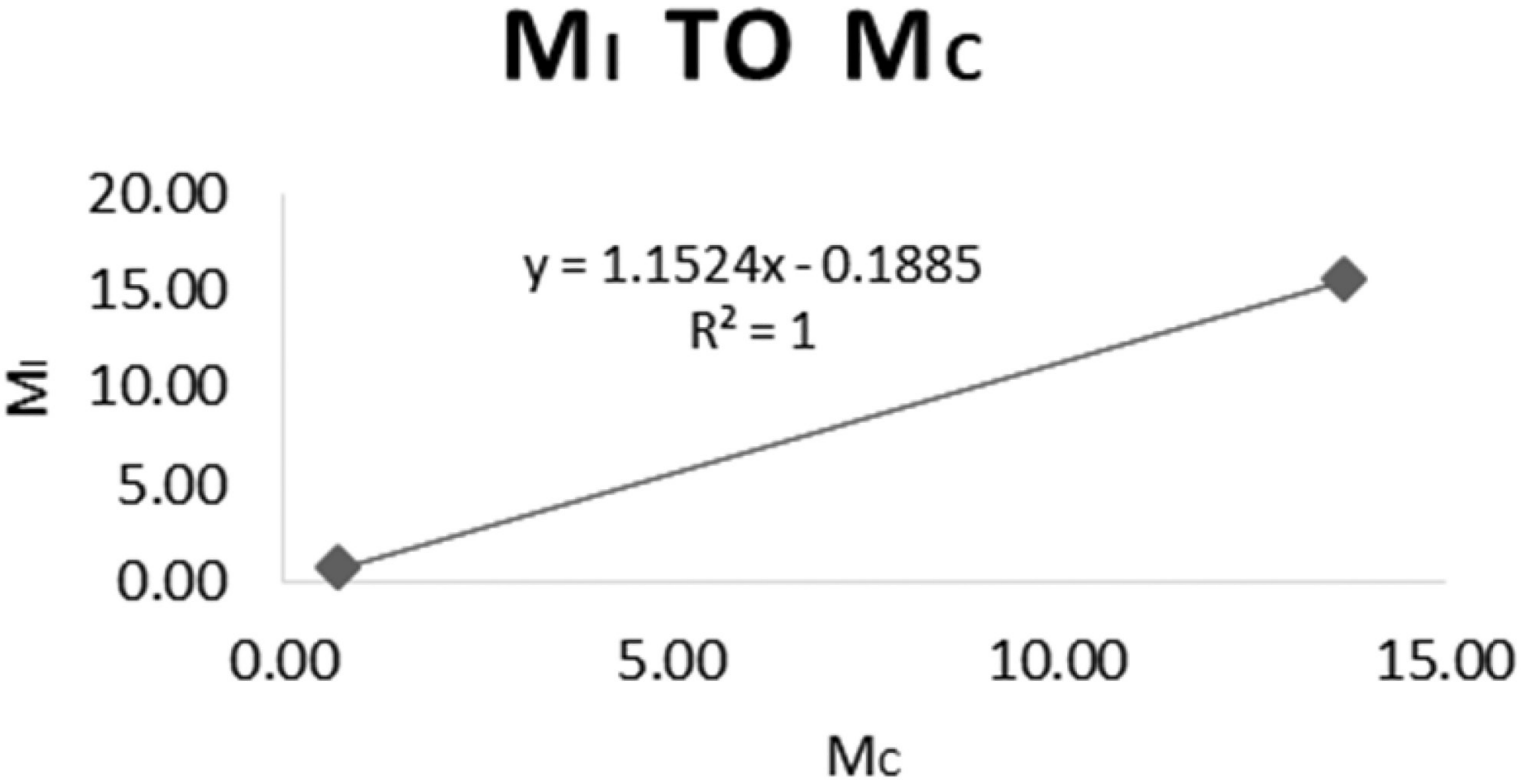
Determining the slope from *M*_i_ to *M*_c_ in the MSI curve.

Also, to obtain the slope for the GAB equation, the correlating data of *M*_i_ and *M*_c_ were plotted (Figure 7).

Based on the results of the plotted charts, the slope of the MSI curve was 1.2486, and the estimated shelf life of the product was 1197.28 days if kept at 30°C and 75% relative humidity. If the relative humidity was set to 73.14, the shelf life would increase to 2003.59 days.

### 3.5 Microbial shelf‐life

The total microbial count on the first day was less than 10 CFU/g, which increased over time. The microbial count at higher storage temperatures was higher than at room temperature.

**FIGURE 8:**
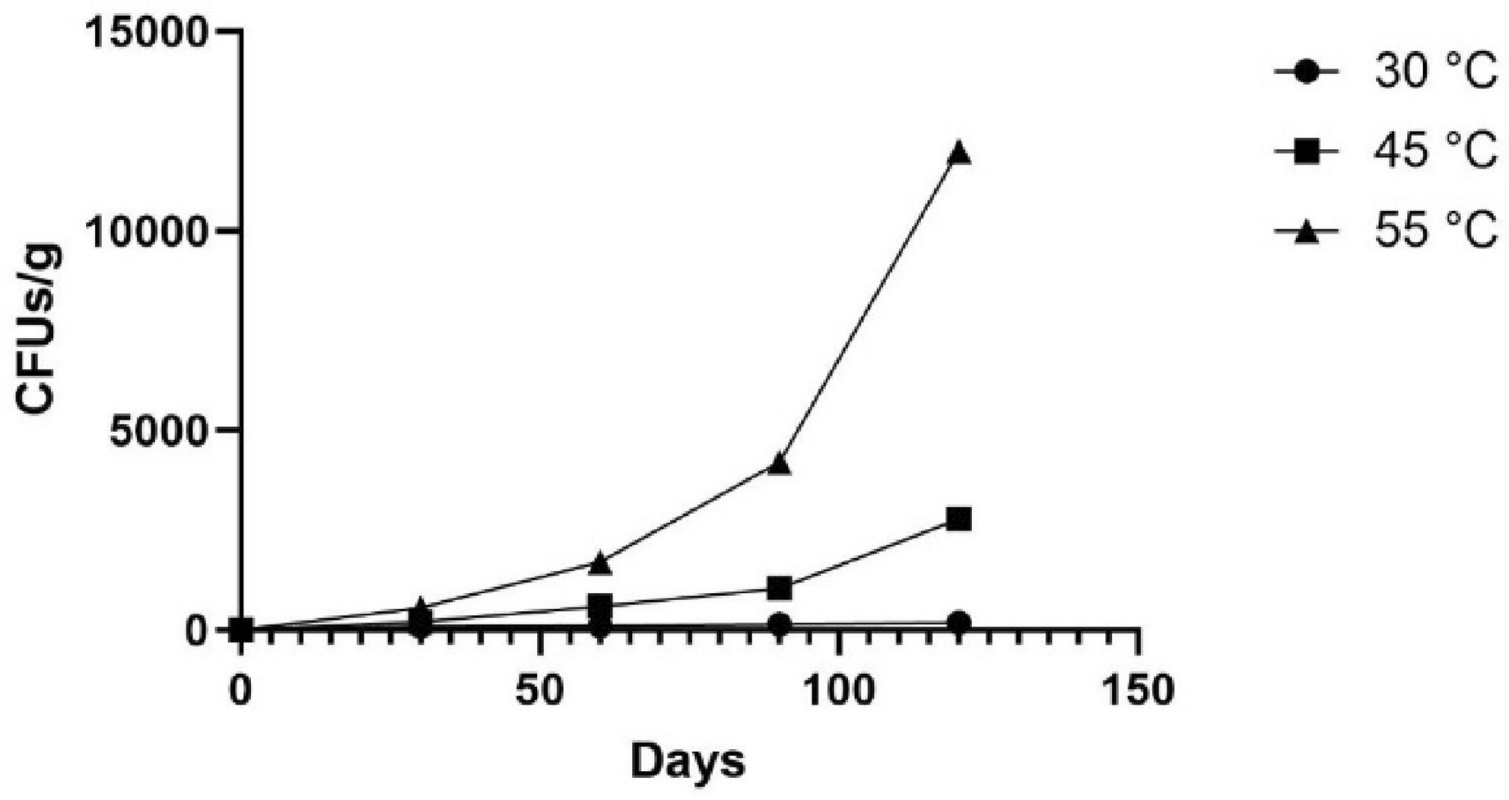
Total microbial count at different storage temperatures.

The fitted equation on the microbial stability curve (Figure 8) was used to estimate the microbial shelf‐life of the formulation. The threshold was set at 100,000 CFUs/g according to similar studies (Tuersuntuoheti et al., 2019; Yu et al., 2023). Based on the curve equations, the microbial shelf‐life estimation at 45 and 55°C is 681.3 and 335.7 days, respectively.

As expected, the total microbial count of the samples increased over time but fell below the threshold in 2 years. The multiple shelf‐life estimations showed that the product can be stored at various temperatures to be used in times of need. The final product's pH measurements show that over time, pH increased more in the samples stored at higher temperatures. This might be associated with the elevated microbial count in these samples.

To increase stability, the water content of the product has to decrease, and preservatives are added occasionally to reduce the risk of microbial growth (Darsch & Moody, 2009; Li et al., 2011). To omit the need to use preservatives, the noodles were fried with beef tallow instead of commercial frying oils because of its iodine value (Hu & Jacobsen, 2016; Shin et al., 2019). Although not measured in this study, other studies suggest the higher stability of tallow compared with commercial vegetable frying oils. Takeoka et al. confirmed that compared with regular vegetable frying oils, tallow had the lowest iodine value and the highest resistance to produce a critical level of polar material, requiring 13 days of 8 h/day heating to produce 25% polar material compared with less than 2 days in cottonseed oil, four in corn oil, and five in canola oil (Takeoka et al., 1997). The iodine value is a measure of the degree of unsaturation in a fat or oil. The higher the iodine value of a fat or oil, the more susceptible it is to oxidation and rancidity (Yang & Boyle, 2016). Unsaturated fatty acids in oils are prone to oxidation, which can lead to the formation of harmful compounds and off‐flavors. Therefore, oils with a higher iodine value tend to have a shorter shelf life and are less stable than those with a lower iodine value (Saad et al., 2008). Animal fats, which are typically more saturated and have a lower iodine value, are more stable and resistant to oxidation (International Organization for Standardization, 2018). It has also been proved that the addition of green extract to tallow can increase its stability (Ardahe & Shahriari, 2020).

Additionally, green tea was also added to the dough mixture instead of water to prevent the fats from oxidation during their shelf‐life. The addition of green tea to the noodle formulation has been proposed by several studies to improve the overall nutrients of the product and benefit from its antioxidant qualities (Kayama et al., 2022; Xu et al., 2019, 2023; Yu et al., 2019, 2020). Green tea contains an amino acid called L‐theanine, which has been shown to promote relaxation and reduce stress (Williams et al., 2020). L‐theanine works by increasing alpha waves in the brain, which are associated with a state of calmness and relaxation (Unno & Nakamura, 2021). Although it contains small amounts of caffeine, which can have a stimulating effect, the L‐theanine helps to counteract this and promote a sense of calm focus. Other compounds in green tea, such as catechins and flavonoids, may also contribute to its calming effects (Cooper et al., 2005; Musial et al., 2020). The state of calmed focus is essential during decision‐making in crises or disasters.

In disasters or battles, one of the most critical factors is time (Barrett & Cardello, 2012). To make the product more porous and hydrophilic to absorb higher amounts of water in less time, CMC was added to the flour. CMC addition has been suggested by several other studies for the same purpose (Jarnsuwan & Thongngam, 2012; Nasruddin et al., 2018; Pongpichaiudom & Songsermpong, 2018; Widaningrum and Haliza, 2022). CMC is a white, indigestible powder that is used in many food and cosmetic products for its properties. CMC molecules absorb water much faster than commercial instant noodles because of their high hydrophilic properties and the tiny porous texture as a result of their incorporation into the flour (Jarnsuwan & Thongngam, 2012).

Inspired by the aforementioned characteristics and other studies on the incorporation of Spirulina in dry noodles (Abd El‐Salam et al., 2017; Agustini et al., 2017; Christwardana et al., 2023; Hadiyanto et al., 2019; Noriko et al., 2020; Riyad et al., 2020; Saad et al., 2008), Spirulina was added to the contents of the final product in its dried form in a sachet. After the heat was lowered, the contents of the sachet would be poured into the dish to benefit the most from its antioxidant contents. It also gives a green and natural look to the prepared meal, making it more palatable to the consumer. Each 7 g of Spirulina contains 4 g of protein, 0.17 mg thiamin, 0.26 mg riboflavin, 0.24 mg pantothenic acid, 0.02 mg vitamin B6, 0.35 mg vitamin E, 8.4 mg calcium, 2 mg iron, 13.6 mg magnesium, 8.26 mg phosphorus, 95.2 mg potassium, 0.14 mg zinc, 73.5 mg sodium, 0.13 mg manganese, and 0.70 mg vitamin C (Seaweed, Spirulina, Dried, FoodData Central Search Results, n.d.).

The estimated shelf‐life period would be detrimental for agencies trying to stockpile such rations for future use, whether in disasters or wars (Feeney et al., 2009; Pahlevan Nejad et al., 2016). The product would have a longer shelf‐life if stored in rather less humid circumstances. The incorporation of antioxidant ingredients such as green tea in the product could have been responsible for the long shelf‐life estimation. However, the most important factor in the shelf‐life of this product has to be the packaging material (Byun et al., 2010; Khan et al., 2014; Li et al., 2011; Sefrienda et al., 2022; Stanley et al., 2019; Zhang et al., 2016). Metal packaging such as aluminum can decrease the risk of water transfer into the package, resulting in a longer shelf‐life (Byun et al., 2010).

## 4 CONCLUSION

Several key ingredients of instant noodle formulations had been replaced in this formulation to enhance its nutritional values and shelf‐life storage, such as wheat flour with semolina flour and soy protein isolate, vegetable oil with beef tallow, or pure water with green tea. The use of Spirulina would improve the nutritional content of the final product. The combination of these alterations with optimum packaging resulted in improved shelf‐stability in accelerated shelf‐life calculations. Thus, the product was shelf‐stable and palatable for enough time to be stored in emergency silos. Considering its enhanced shelf‐stability and nutritional contents, it is recommended to use this product in disaster conditions.

## AUTHOR CONTRIBUTIONS

**Farboud Shahabinejad:** Conceptualization (equal); data curation (equal); formal analysis (equal); funding acquisition (equal); investigation (equal); methodology (equal); project administration (equal); resources (equal); software (equal); supervision (equal); validation (equal); visualization (equal); writing – original draft (lead); writing – review and editing (equal). **Maryam Ghorbani:** Conceptualization (equal); data curation (equal); formal analysis (equal); funding acquisition (equal); investigation (equal); methodology (equal); project administration (equal); resources (equal); software (equal); supervision (equal); validation (equal); visualization (equal); writing – original draft (equal); writing – review and editing (equal). **Sepideh Abbaszadeh:** Conceptualization (equal); data curation (equal); formal analysis (equal); funding acquisition (equal); investigation (equal); methodology (equal); project administration (equal); resources (equal); software (equal); supervision (equal); validation (equal); visualization (equal); writing – original draft (equal); writing – review and editing (equal). **Mohammad Nejatian:** Conceptualization (equal); data curation (equal); formal analysis (equal); funding acquisition (equal); investigation (equal); methodology (equal); project administration (equal); resources (equal); software (equal); supervision (equal); validation (equal); visualization (equal); writing – original draft (equal); writing – review and editing (equal). **Maryam Taghdir:** Conceptualization (equal); data curation (equal); formal analysis (equal); funding acquisition (equal); investigation (equal); methodology (equal); project administration (lead); resources (equal); software (equal); supervision (lead); validation (lead); visualization (equal); writing – original draft (equal); writing – review and editing (equal).

## ACKNOWLEDGEMENTS

The authors would like to thank the emergency relief agencies who provided their insights on the development of the formulation.

## FUNDING INFORMATION

There is no financial support related to this work.

## CONFLICT OF INTEREST STATEMENT

All authors have declared no conflict of interest.

## ETHICS STATEMENT

The study was approved by the ethics committee for Research at Baqiyatallah University of Medical Sciences (IR.BMSU.REC.1400.106).

## Data Availability

The data that support the findings of this study are available on request from the corresponding author.
